# Metabolo-epigenetic interplay provides targeted nutritional interventions in chronic diseases and ageing

**DOI:** 10.3389/fonc.2023.1169168

**Published:** 2023-06-19

**Authors:** Marta Gómez de Cedrón, Rocío Moreno Palomares, Ana Ramírez de Molina

**Affiliations:** ^1^ Molecular Oncology Group, IMDEA Food Institute, CEI UAM, CSIC, Madrid, Spain; ^2^ Cell Metabolism Unit, IMDEA Food Institute, CEI UAM, CSIC, Madrid, Spain; ^3^ FORCHRONIC S.L, Avda. Industria, Madrid, Spain

**Keywords:** epigenetics, chronic diseases, bioactive compounds, metabolism, ageing

## Abstract

Epigenetic modifications are chemical modifications that affect gene expression without altering DNA sequences. In particular, epigenetic chemical modifications can occur on histone proteins -mainly acetylation, methylation-, and on DNA and RNA molecules -mainly methylation-. Additional mechanisms, such as RNA-mediated regulation of gene expression and determinants of the genomic architecture can also affect gene expression. Importantly, depending on the cellular context and environment, epigenetic processes can drive developmental programs as well as functional plasticity. However, misbalanced epigenetic regulation can result in disease, particularly in the context of metabolic diseases, cancer, and ageing. Non-communicable chronic diseases (NCCD) and ageing share common features including altered metabolism, systemic meta-inflammation, dysfunctional immune system responses, and oxidative stress, among others. In this scenario, unbalanced diets, such as high sugar and high saturated fatty acids consumption, together with sedentary habits, are risk factors implicated in the development of NCCD and premature ageing. The nutritional and metabolic status of individuals interact with epigenetics at different levels. Thus, it is crucial to understand how we can modulate epigenetic marks through both lifestyle habits and targeted clinical interventions -including fasting mimicking diets, nutraceuticals, and bioactive compounds- which will contribute to restore the metabolic homeostasis in NCCD. Here, we first describe key metabolites from cellular metabolic pathways used as substrates to “write” the epigenetic marks; and cofactors that modulate the activity of the epigenetic enzymes; then, we briefly show how metabolic and epigenetic imbalances may result in disease; and, finally, we show several examples of nutritional interventions - diet based interventions, bioactive compounds, and nutraceuticals- and exercise to counteract epigenetic alterations.

## Graphical Abstract

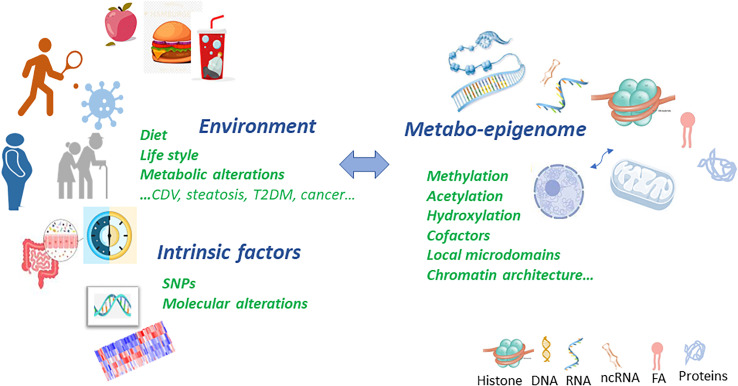

## 1 Interplay between cellular metabolism and epigenome

Epigenetics refers to heritable changes in gene expression that are not caused by DNA sequence alterations. Although epigenetics is implicated in cell fate stability in multicellular organisms, it is highly affected by short-term environmental inputs such as stress conditions or nutrient supply (1). Alterations in the epigenome have been associated to a wide variety of disease related processes including non-communicable diseases (NCD), cancer (considered as chronic disease when it can be controlled with treatment, becoming stable and/or reaching remission) and ageing (2, 3). According to the World Health Organization (WHO), 80% of heart diseases, strokes, type 2 diabetes and over a third of cancers can be prevented by modifying life-style factors, such as cutting out tobacco, eating a healthy diet, being physically active and stopping harmful use of alcohol (4). As diet and exercise can modify the metabolic disease risk, it is crucial to understand how life-style factors contribute to shape the epigenome and, subsequently, how alterations in the epigenetic landscape contribute to disease. Indeed, the epigenome is rapidly affected by the availability of central metabolites that can be used as either substrates or allosteric cofactors, fine-tuning the activities of the epigenetic enzymes (5, 6). For this reason, diet based interventions, including nutraceuticals and bioactive compounds, and exercise may provide therapeutic tools to restore a “healthy” epigenome against metabolic diseases (7).

Life-style factors, including diet and physical activity, are the main source of metabolites, which provide the chemical moieties for DNA, RNA and histone modifications, and cofactors, which modulate the activity of epigenetic enzymes-. Indeed, central cellular metabolites, such as S-adenosyl methionine (aCETY), acetyl-coenzyme A (acetyl-CoA), adenosine triphosphate (ATP), nicotinamide adenine dinucleotide (NAD^+^) and flavin adenine dinucleotide (FAD) are key sensors of nutrient availability and they contribute to the regulation of gene expression by epigenetic mechanisms (8). On the other hand, epigenetics contributes to regulate metabolism by affecting gene expression. Thereby, a crosstalk between epigenetics and metabolism exists to determine molecular programs.

Over the past 20 years, there has been a great interest on the identification and characterization of enzymes responsible for adding or removing epigenetic marks. The activity of many of these chromatin-modifying enzymes -DNA methyltransferases (DNMTs), DNA hydroxylases (DNHDs), histone acetyltransferases (HATs), histone deacetylases (HDACs), histone methyltransferases (HMTs), and histone demethylases (HDMs)- is regulated partially by the concentrations of their required metabolic substrates or cofactors (9). Chromatin plays important roles in DNA biology including gene expression regulation. The level of chromatin compaction has important consequences for gene transcription as it influences the accessibility of DNA sequences to transcription factors and other regulatory proteins. Thus, modifications on DNA and histones regulate the level of chromatin compaction either directly or by facilitating the binding of remodeling proteins that recognize modified sites. Modifications of RNA (epitranscriptomics) add an additional level of gene expression regulation and might influence RNA transport, splicing, stability, and translation. Furthermore, non-coding RNA sequences (mainly microRNAs and long non-coding RNAs) have been extensively shown to play a key role in the regulation of gene expression.

Nowadays, it is well established that NCD and ageing are affected by the interaction of life-style factors -diet and exercise-, metabolism and the epigenome. As such, the availability of metabolites is central for the epigenetic changes, partially determining differentiation programs, cell identity, stemness, functional plasticity and environmental responses (10).

Plasticity is crucial for all biological functions, and epigenetics is implicated in the adaptation to multiple signals and conditions by mean of several mechanisms (11, 12):

(1) Changes in the levels of cellular metabolites modulate the epigenome translating the metabolic state of a cell into changes of the chromatin pattern. For example, high levels of acetyl-CoA facilitate the acetylation of histone and non-histone proteins of transcriptional complexes (TC); high levels of NAD^+^ activate sirtuin-HDACs to promote transcriptional silencing; high glucose levels may increase the synthesis of UDP-GlcNac by the hexosamine pathway, stimulating the activity of the MLL5 methyltransferase to increase GlcNAcylation (13).

(2) DNA-binding factors recruit histone-modifying enzymes to specific chromosomal domains to stimulate their enzymatic activity locally. This is the case of Mat IIIa, implicated in the synthesis of SAM, for H3K9-specific histone methyltransferases to repress transcription (14).

(3) Enzymes that use the same metabolite, such as DNA or histone methyltransferases, may compete with each other resulting into distinct methylation products (15).

(4) Global and local fluctuations in the concentration of cofactors also modulate the activity of epigenetic enzymes. The relative activity of the three sirtuins present in the nucleus (SIRT1, SIRT6, and SIRT7) may modulate the local concentration of NAD^+^, thereby resulting in the reciprocal regulation of the other sirtuins localized nearby chromatin microdomains.

(5) Epitranscriptomic modifications, including the *N* ^6^-methyladenosine (m^6^A), have been shown to regulate the processing, translation, and stability of mRNAs; thereby influencing cell biology (16). Posttranscriptional epigenetic modification of RNA (the so-called ‘epitranscriptome’) has emerged as a fascinating field of research (for a detailed discussion on this topic, readers can refer to several excellent reviews (17–19).

As indicated, metabolism mainly modulates the activity, the localization of chromatin remodelling enzymes, and the levels of substrates and cofactors used by chromatin modifying enzymes. For example, chromatin regulators can directly recognize specific patterns of DNA sequence, such as the percentage of GC (20), or can be affected by the local levels of specific metabolites. Although a vast network of central metabolites are substrates for chromatin regulators, such as histone and DNMTs (21), some others can bind to chromatin proteins affecting the cell metabolism and function. A recent study showed a metabolic link between the macrodomain of H2A1.1 histone regulating the mitochondrial metabolism and cell function by sequestering the NAD^+^ metabolite for ADP-ribose and ADP-ribosylated proteins (22, 23).

Here, we first describe key metabolites derived from the cellular metabolic pathways that are used as substrates -to “write” the epigenetic marks-; and cofactors that modulate the activity of epigenetic enzymes; then we briefly show how metabolic and epigenetic imbalances result in disease; and finally, we show several examples of nutritional interventions - diet based interventions, bioactive compounds, and nutraceuticals- and exercise to counteract epigenetic imbalances.

## 2 Metabolic regulations of epigenetic marks

### 2.1 Metabolic regulation of DNA methylation

DNA methylation is one of the most vastly studied epigenetic marks that determines gene expression profiles, as well as germline imprinting. However, balanced DNA methylation is essential to coordinate these processes. To achieve DNA methylation balance, both, DNA methyltransferase enzymes and the TET (ten eleven translocation) family of dioxygenases mediate the deposition and removal of methyl groups on DNA, respectively.

DNA methyltransferase enzymes -DNMT1, DNMT3a, and DNMT3b- generate 5-methylcytosine (^5^mC), which is a major transcriptional repressive mark in many eukaryotes (24, 25). DNMT1 is mainly implicated in the germline imprinting (26) meanwhile “*de novo*’’ methyltransferases (27) are more prone to be activated in response to intracellular metabolic changes. On the contrary, demethylation of ^5^mC is driven by the TET family of dioxygenases that oxidize ^5^mC to form 5-hydroxymethylcytosine (^5^hmC) which can be further oxidized to form carbonylmethylcytosine (camC) and formylmethylcytosine (fmC), or glycosylated (28). The activity and expression levels of cytosine methyltransferases are also affected by specific epigenetic marks and post-translational modifications such as the histone methylation H3K4, H3K36 (29), highlighting the complexity of the epigenetic network.

**Figure 1:**
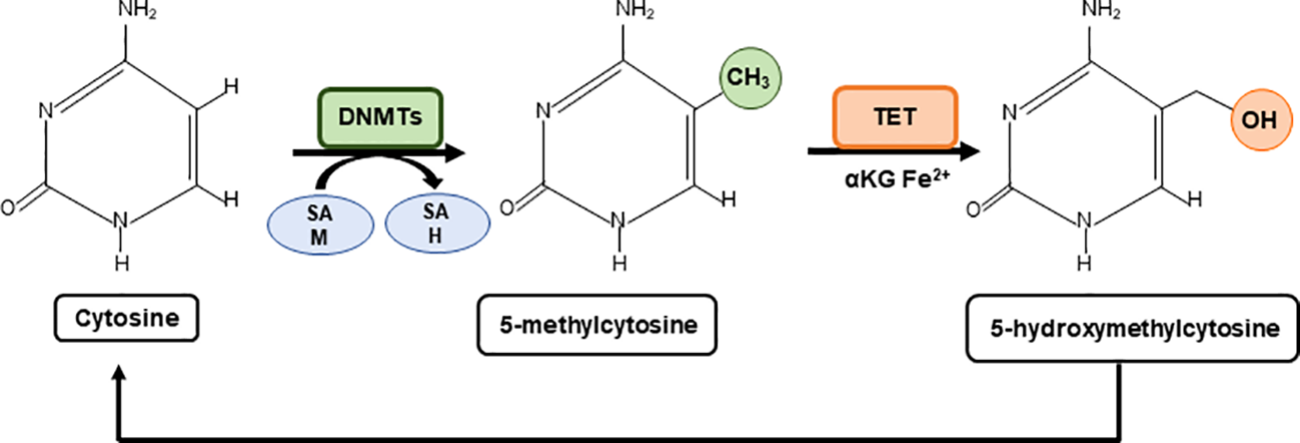
Metabolic regulation of cytosine methylation. A family of DNA methyltransferases (DNMTs) catalyse the transfer of a methyl group from S-adenylmethionine (SAM) to the fifth carbon of cytosine residue to form 5-methylcytosine (5mC) producing S-adenosyl-L-homocysteine (SAH). The opposite process is carried out by ten-eleven-translocation (TET) proteins which use α-Ketoglutarate (α-KG) and Fe^2+^ as cofactors to generate 5-hydroxymethylcytosine (5hmC). 5hmC can be further oxidised to form carbonylmethylcytosine (camC) and formylmethylcytosine (fmC), or glycosylated (not shown).

The methylation and the demethylation of cytosines use central metabolites as substrates such as the one carbon donor SAM for methylation, and alpha ketoglutarate (α-KG) and Fe^2+^ for TET mediated demethylation, which is enhanced by ascorbate (vitamin C) (30). Thus, the levels of these metabolites shape the cytosine methylation profile with downstream effects on gene expression (**Figure 1**).

Intracellular cell signalling pathways drive rapid epigenetic mechanisms through metabolic manipulation to restore cellular homeostasis. In this line, activated AMPK favours the increase levels of α-ketoglutarate (αKG) to activate the TET-dependent demethylation of the *PRDM16* promoter during brown adipogenesis (31). Interestingly, dietary α-KG promotes beige adipogenesis to prevent obesity in middle-aged mice (32), indicating the therapeutic potential of diet to shape the altered epigenome in metabolic diseases.

Increased levels of α-KG -after the administration of glucose, glutamate or glutamine- correlate with a rapid increase in ^5^hmC levels in the liver of mice (28). Similarly, supplementation of vitamin C in the extracellular medium of embryonic stem cells (ESC) induces a global increase in cytosine hydroxymethylation, at the expense of the ^5^mC (33), leading to a rapid reprogramming of fibroblasts into induced pluripotent stem cells (iPSCs). In cancer, a global hypomethylation epigenetic profile, together with inter-dispersed hypermethylated CpG islands (CIMP), constitutes a prognostic biomarker in multiple tumours (34). A specific point mutation in the isocitrate dehydrogenase (*IDH1*) gene in gliomas results in a mutant enzyme that generates high levels of the oncometabolite 2-hydroxyketoglurate, inhibiting α-KG-dependent TET enzymes (35).

### 2.2 Metabolic regulation of RNA methylation

RNA modifications can also affect mRNA splicing, stability, nuclear transport, and translation (36). The main RNA modification is the methylation of adenosine at position 6 to produce m^6^A, which is catalyzed by the methyltransferase-like 3 (METTL3)–METTL14 complex. This epigenetic mark can be removed by non-heme-Fe(II)/2-oxoglutarate(2OG)-dependent oxygenases, fat mass and obesity-associated protein (FTO) and the ALKN homolog 5 (ALKBH5) (37). Increasing evidence has shown that enzymes implicated in the m^6^A remodeling are deregulated in chronic diseases, ageing and cancer (38, 39). For example, METTL3 is upregulated in some tumors inducing proliferation and dissemination, and it has been proposed as a therapeutic target (40).

Thus, there is a great interest in modulating the activity of enzymes implicated in m^6^A remodeling. In this regard, inhibitors of FTO have been shown promising results in preclinical models of myeloid leukaemias and glioblastomas (41). Moreover, FTO inhibitors have been demonstrated to improve insulin sensitivity in high fat diets (HFDs) induced obesity (42).

### 2.3 Metabolic regulation of histone methylation

Histone methylation/demethylation, similar as DNA methylation, is regulated by cellular levels of SAM, Fe^2+^/Fe^3+^ and α-KG. Histone methyltransferases (EZH2, G9A) utilize SAM as a methyl donor and histone demethylases, JmjC-family of histone demethylases and LSD-family of histone demethylases (LSDs), employ α-KG as cofactor for their enzymatic reactions.

While ^5^mC on DNA is a major transcriptional repressive mark in many eukaryotes, the effect of histone methylation in gene expression depends on the type of residue and the number of methyl groups added. Generally, methylation of H3K9, H3K27, and H4K20 are associated to silenced chromatin; on the contrary, H3K4, H33K36, H3K48, and H3K79 marks are associated to active gene expression (43). α-KG cofactor is required for the demethylation reactions of JmjCs, while LSDs require the FAD cofactor to demethylate the histone residues (6).

Glucose and glutamine catabolism are used to maintain a high α-KG/succinate ratio, enhancing the global demethylation of multiple histone residues (44). For this reason, manipulation of the α-KG/succinate ratio can balance the renewal *versus* differentiation fate in ESC cultures. Similarly, in adipocytes, various promoters of genes involved in energy expenditure, such as *PGC-1A* and pyruvate dehydrogenase kinase 4 (*PKD4*), are demethylated by the histone demethylase LSD1 (KDM1A), and, for this reason, their expression is dependent on the availability of intracellular FAD (45).

### 2.4 Metabolic regulation of histone acetylation

A rapid response to nutrient availability may also be mediated by the acetylation or deacetylation of histones (46). HATs use the substrate acetyl-CoA, which is produced by several catabolic pathways - glycolysis, fatty acid β-oxidation, and amino acid catabolism-. It has been described that high levels of acetyl-CoA augments the overall levels of histone acetylation to promote the expression of genes related to adipocyte differentiation (47).

While histone acetylation naturally relies on acetyl-CoA, a subset of HDACs use NAD^+^ as a cofactor. This feature is unique to the sirtuin family of HDACs, which cleave NAD^+^ to generate nicotinamide and 1-O-acetyl ADP-ribose (OAR) during histone deacetylation (48). The increase ratio of NAD^+^/NADH is another energy sensor for the class III histone deacetylases sirtuins, repressing the expression of genes involved in lipogenesis, glycolysis and gluconeogenesis (49, 50). On the contrary, when glucose is abundant the ratio of NAD^+^/NADH is diminished reducing the activity of sirtuins to favour lipogenesis and gluconeogenesis. In addition, pyruvate derived from glycolysis is used to fuel the tricarboxylic acid (TCA) augmenting the levels of acetyl-CoA and, consequently, modulating the activity of HAT (51). All in all, the metabolic control of sirtuins and HAT may provide a promising field of research affecting not only gene expression but also the mitochondrial metabolism.

### 2.5 Other epigenetic marks regulated by metabolism

In addition, other epigenetic modifications can be regulated by metabolism. There is a wide range of post-translational modifications (PTMs) on histone and non-histone proteins, integrating environmental changes into cellular responses by regulating gene expression. This is the case of acylation, lactylation, 2-hydroxy-isobutyrylation, succinylation, and malonylation of targeted proteins, which may affect cell function, proliferation, and differentiation. Histone modifications is an important type of epigenetic modification that has been widely detected and exerts different effects at different sites (52–54).

In 2019, Zhang et al. discovered a brand new epigenetic modification called lactylation on lysine residues affecting gene transcription from chromatin (55). The glycolytic-dependent metabolism found in tumours and fast-growing cells has made lactate a pivotal player in energy metabolism reprogramming, which enables cells to obtain abundant energy in a short time. Moreover, lactate shapes the acidic tumor microenvironment, recruiting immunosuppressive cells in cancer. Recently lactate-induced lactylation has been shown to modify histone proteins to alter the spatial configuration of chromatin, affect DNA accessibility and regulate the expression of corresponding genes (55). Thus, in non-small cell lung cancer cells, lactate attenuates glucose uptake, which seems to be partially affected by the increased histone lactylation in the promoters of the glycolytic enzymes (HK-1 and PKM) (56). In macrophages, lactate was reported to induce histone lactylation in the promoters of the profibrotic genes, which is concordant with the upregulation of this very epigenetic modification in the macrophages in fibrotic lungs. Moreover, the role of histone lactylation in tumorigenesis is gradually found, just as the oncogenic role found in other histone modifications (55).

## 3 Metabolic regulation of non-coding RNAs

Non-coding RNAs (ncRNAs) are RNA molecules which are not translated into proteins but post-transcriptionally regulate gene expression. The most functionally relevant types of non-coding RNAs studied in the context of chronic diseases are microRNAs (miRNAs) and long non-coding RNAs (lncRNAs).

ncRNAs modulate key metabolic pathways including hypoxia-inducible pathways, glycolysis, oxidative phosphorylation, lipid metabolism, amino acid metabolism and signal transduction pathways, among others. Thus, they may be biomarkers for prognosis, and personalized therapeutic interventions.

### 3.1 microRNAs

MicroRNAs -small molecules of RNA (≈22 nucleotides)- are well known regulators of adipogenesis, fatty acid metabolism, insulin signalling, inflammation, and cell development and differentiation.

Several miRNAs, such as miR-30, miR-26b, miR-199a, and miR-148a- are differentially expressed in obese people, being proposed as biomarkers of metabolic health. miR-17-5p and miR-132- are overexpressed in visceral inflammatory adipose tissue correlating with body mass index, glycosylated hemoglobin, diabetes, and dyslipidaemia.

Metabolic alterations, such as obesity, insulin resistance, ageing and cancer, affect microRNA expression. For example, miR21 is induced during obesity correlating with impaired vascular function (57). On the contrary, silencing this microRNA has been demonstrated to reduce adipogenesis and triglyceride accumulation, improving insulin sensitivity and reducing systemic inflammation.

Different studies reported that the expression of miRNAs is directly correlated with diet and lifestyle, being miR-17/20/93 family, miR-21/590-5p family, miR-200b/c family, miR-221/222 family, let-7/miR-98 family and miR-203 associated with specific diets.

The expression of miR-21 was found diminished in the white adipose tissue (WAT) of obese humans correlating with BMI (58). Interestingly, locked nucleic acid (LNA)-miR-21 treatment reduced body weight (59) in a preclinical model of diet-induced obesity (DIO). In another study, let-7 knockout mice did not develop insulin resistance after a high fat diet induced obesity (60).

In a clinical trial with obese women, reduction of body weight showed that circulating levels of several miRNAs were similar to lean controls, suggesting that some of them could be used as biomarkers (61).

### 3.2 LncRNAs

More research has recently begun to unravel the biological functions of lncRNAs, which are tissue-specific long RNA transcripts (>200 bp). GYG2P1, lncRNAp21015, and lncRNA-p5549 are examples of lncRNAs that are differentially expressed in obesity (62). Their expression has been found inhibited in obese individuals. RP11-20G13.3 is among the lncRNAs required to maintain PPAR, C/EBP, and ADIPOQ levels during adipogenesis and is differentially expressed in obesity (63). Other lncRNAs, such as lnc-dPrm16 and MIST, have been shown to influence brown adipogenesis, inflammation, and lipid metabolism (64).

## 4 Intracellular metabolic pathways implicated in the regulation of epigenetics

### 4.1 SAM and the one-carbon metabolism pathway (folate and methionine cycles)

A central methylation pathway in eukaryotic cells is the *one-carbon metabolism*, implicated in the transfer of methyl groups to various substrates and cofactors within the folate and methionine cycles. The transfer of methyl groups from 5-methyltetrahydrofolate (5-mTHF) to homocysteine (Hcy) to form methionine connects the two cycles. Methionine is then converted to SAM, the universal methyl donor for DNA, proteins, and secondary metabolites. Methyltransferases use SAM as donor of methyl groups, and the resulting demethylated *S*-adenosyl-l-homocysteine (SAH) is hydrolyzed to form Hcy, which is then recycled to methionine using 5-MTHF as a methyl group donor, thus completing the cycle. Nicotinamide N-methyltransferase (NNMT) consumes methyl donors and makes SAM unavailable to other methyltransferases, thereby repressing H3K27me3 marks. NNMT is involved in the maintenance of the naïve state of ESC which are characterized by low levels of histone methylation (65, 66).

Both HMTs and DNMTs use SAM as a donor for the methylation reactions. Importantly, the ratio of SAM/SAH is an indicator of the status of the one-carbon metabolism to regenerate methionine (67).

Nutrients such as methionine, folate, choline, betaine, and vitamins B2, B6, and B12 are precursors of SAM affecting the SAM/SAH ratio and the global levels of DNA methylation. However, it is difficult to extrapolate the direct role of metabolism on DNA methylation, because the mechanisms regulating from DNA methylation are very complex and the relationship between methylation pattern and gene expression is tissue and context-dependent (68).

The balance of methionine, folate, and B12 from our diet regulates the activity of the folate and methionine cycles, which are mechanistically co-dependent. The *folate cycle* converts tetrahydrofolate (THF), into 5,10-methylene-THF by serine hydroxymethyltransferase (SHMT), an enzyme that requires B6 as a cofactor. Then, 5,10-methyleneTHF acts as a methyl-donor for thymidylate synthase (TS) to synthesize deoxythymidine monophosphate (dTMP) from deoxyuracil monophosphate (dUMP) or for methylenetetrahydrofolate reductase (MTHFR) to generate 5-mTHF (69). In the *methionine cycle*, methionine is converted to SAM by the activity of methyl-adenosyl transferase 2A (MAT2A). SAM is then used as a methyl donor resulting in S-adenosylhomocysteine (SAH), which can be further hydrolyzed to Hcy by S-adenosylhomocysteine hydrolase (AHCY). HCy is then converted into methionine by methionine synthase (MS) using 5-mTHF as a methyl donor and vitamin B12 as an essential cofactor (70). In addition, methionine can also be recycled from Hcy using betaine, derived from dietary choline, as a methyl donor (71), or directly from SAM through the polyamine synthesis and the methionine salvage pathway. Using vitamin B6 as a cofactor, Hcy can also be converted to cysteine for the synthesis of glutathione (72).

Vitamin B12, in addition to its role as a cofactor in the recycling of Hcy to methionine, is an important cofactor for the metabolic enzyme methyl-malonyl CoA mutase (MUT), which is located in mitochondria. MUT uses vitamin B12 in the form of adenosylcobalamin to convert L-methylmalonyl-CoA to succinyl-CoA, which can further enter the TCA cycle. Genetic loss of MUT activity can disrupt TCA cycle function and mitochondrial redox function. Thus, the levels of vitamin B12 connect the one-carbon metabolism, energy metabolism, and redox metabolism (73).

### 4.2 Glycolysis, glutaminolysis and fatty acid oxidation pathways

Glycolysis, glutaminolysis and fatty acid oxidation provide different metabolites that are key for epigenetic modifications. These pathways provide metabolic cofactors for epigenetic reactions and interact with each other in a complex crosstalk that links metabolic needs to gene expression responses.

#### 4.2.1 Glycolysis

Glycolysis is a key metabolic pathway implicated in the production of metabolites such as pyruvate, intermediates of the pentose phosphate pathway (PPP) and the one carbon metabolism, among others. Pyruvate may be subsequently metabolized to lactate by the enzymatic activity of lactate dehydrogenase (LDHA) or converted to acetyl-CoA through the pyruvate dehydrogenase enzyme (PDH), which enters the mitochondria to feed the TCA cycle. Interestingly, both lactate and acetyl-CoA are crucial donors of the epigenetic modifications including histone lactylation (74) and histone acetylation (75).

The PPP contributes to the synthesis of nucleotides for cell division and contribute to generate the methyl donor SAM (76).

The glycolytic flux determines the ratio of NAD+/NADH ratio which is important for the activities of sirtuin HDACs.

Cellular glucose availability is linked to acetyl-CoA abundance and histone acetylation. The uptake of glucose is mainly driven by the glucose transporters type 1 and 4 (GLUT1/4), which are frequently upregulated in activated T cells and cancer cells to support cell proliferation (77). Pyruvate decarboxylase complex (PDC) produces acetyl-CoA, connecting aerobic glycolysis to TCA cycle. LDHA produces lactate from pyruvate in highly proliferative cells where an aerobic glycolysis switch occurs over mitochondrial oxidative phosphorylation (78). The enzyme PDC is found in both mitochondria and the nucleus. Nuclear PDC regulates the expression of sterol regulatory element-binding transcription factor (SREBP) target genes by affecting histone acetylation, and mitochondrial PDC contributes to the production of cytosolic citrate for lipogenesis (79). The availability of acetyl-CoA for HATs is also modulated by the levels of GLUT1/4 -implicated in the uptake of glucose (77)- and the levels of ATP- citrate lyase (ACLY) (80), which are frequently upregulated in activated T cells and cancer cells to support cell proliferation (81).

Glucose is the main carbon source for acetyl-CoA in oxygenated cells. An exquisite mechanism is observed in cancer cells during hypoxia to promote the expression of lipogenic genes acetyl-CoA carboxylase alpha (*ACACA*) and fatty acid synthase (*FASN*), by mean of the acetate mediated histone H3 acetylation. The acetyl-CoA synthetases ACSS1/2, which catalyze the production of acetyl-CoA from acetate, contribute to the above acetate-mediated epigenetic regulation (82).

Acetyl-CoA levels change with nutrient abundance and for this reason it can be considered as a nutrient sensor to respond to metabolic changes (83). For example, acetyl-CoA is produced from distinct metabolites such as pyruvate, citrate and acetate which can be produced by the activity of the PDC, ACLY and ACSS2, respectively. In addition, acetyl-CoA levels depend on the levels of ketone bodies and fatty acid *β*-oxidation (84).

#### 4.2.2 Glutaminolysis

Glutaminolysis is a metabolic pathway that generates α-KG which, as seen above, is a cofactor for dioxygenases and histone, DNA and RNA demethylases. Glutaminolysis is required for the glutamine- dependent anaplerosis of TCA providing metabolic substrates such as α-KG. Similarly, other TCA metabolites including succinate, fumarate, malate and oxalacetate are relevant epigenetic modulators as they can inhibit cellular demethylases due to their similar chemical structure to α-KG. Furthermore, the α-KG derived metabolite 2-hydroxyglutarate (2-HG) has been shown to interfere with the activity of demethylases and thus the global epigenetic marks (85). Several TCA cycle intermediates can be exported out of mitochondria including citrate and α−KG. Cytosolic citrate is converted to acetyl-CoA, which is used as a donor for HAT-mediated histone acetylation. α−KG is used as cofactor for histone and DNA demethylation reactions by JHDM and TET, respectively.

The substrate for HMT and DNMT is SAM, which is synthesized from the essential amino acid methionine The propionate catabolic pathway breaks down branched-chain amino acids (BCAAs), odd-chain fatty acids, and cholesterol to be used in the TCA cycle in the mitochondria. MUT converts methylmalonyl-CoA to succinyl-CoA using vitamin B12, in the form of adenosylcobalamin, as a cofactor. Succinyl-CoA then enters the TCA cycle.

#### 4.2.3 Fatty acid oxidation

Fatty acid oxidation (FAO) is a key metabolic pathway affecting the levels of NAD^+^/NADH and FAD/FADH_2_ metabolites, which are key cofactors of epigenetic enzymes. In addition, augmenting FAO increases the levels of acetyl-CoA which will affect the acetylation levels of histones (86). Acetyl-CoA can also enter in the TCA to produce citrate. In the cytosol, citrate is converted to acetyl-CoA and malonyl-CoA by the ACLY enzyme. ACCS1/ACCS2 catalyze the conversion of acetyl-CoA to malonyl-CoA, which is the rate-limiting step for the synthesis of FAs. ACCs are activated by citrate and inhibited by palmitoyl-CoA and malonyl-CoA. When there are low levels of energy, AMPK phosphorylates ACCs to increase FAO in the short term. In addition, diet affects the expression of genes implicated in the synthesis of FAs or in FAO (87).

Although changes in global availability of metabolites impact on epigenetic processes globally (e.g. SAM levels regulate methylation), the enzymatic Km of the enzymes for those metabolites, and the availability of other micronutrients, such as vitamins and minerals, will fine-tune the epigenetic changes upon metabolic alterations (88). Thus, in each metabolic context, specific epigenetic marks may be favored. For this reason, balanced metabolic traits are necessary to achieve coordinated epigenetic responses that sustain health.

**Figure 2:**
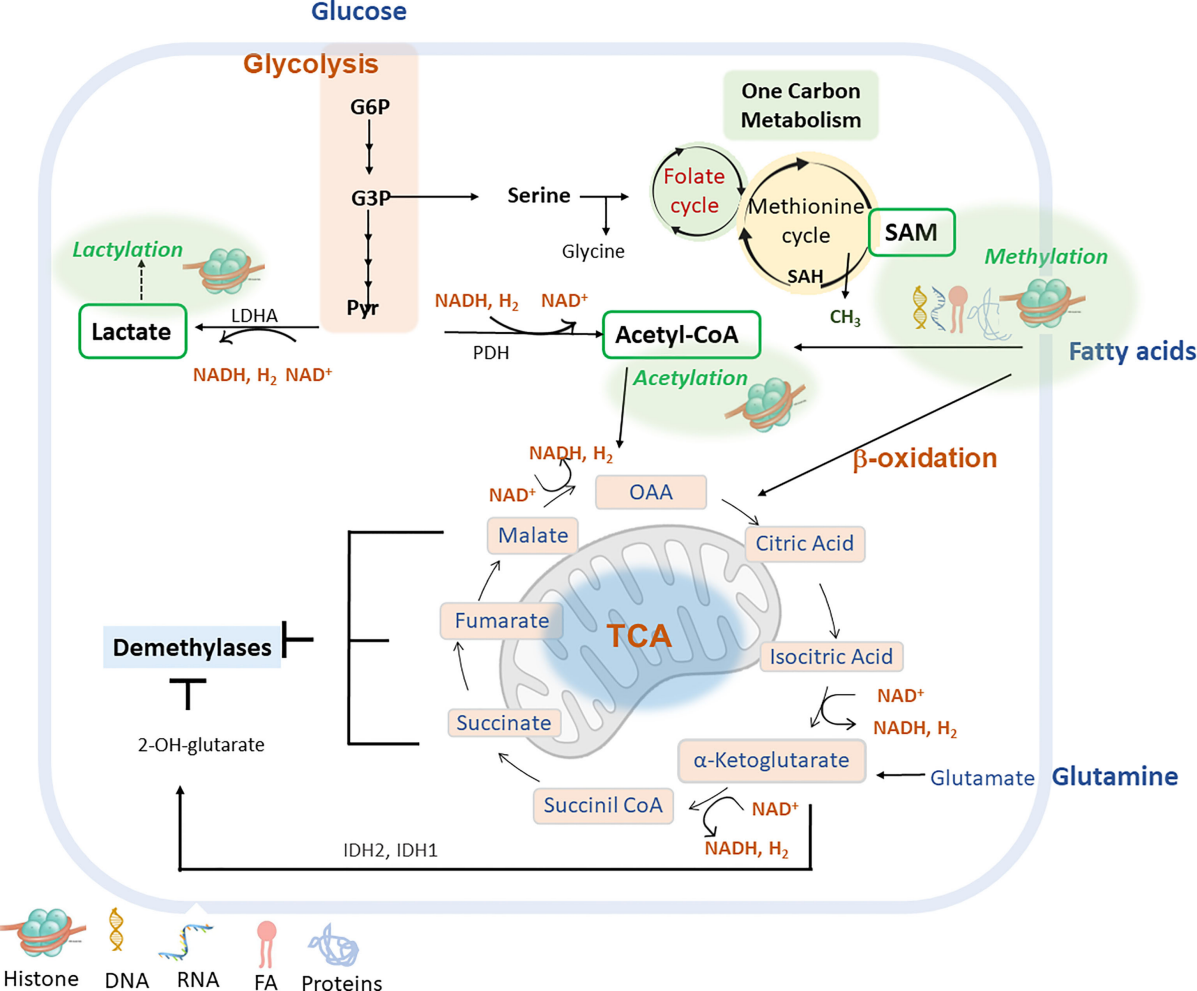
Main metabolic pathways can be implicated in the epigenetic remodelling include glycolysis, fatty acid β-oxidation (FAO), glutaminolysis and lactylation via derived metabolites and cofactors (lactate, acetyl-CoA, α-ketoglutarate or NAD^+^). G6P (glucose-6-phosphate); G3P (glycerol-3-P); Pyr (pyruvate); LDHA (lactate dehydrogenase A); PDH (pyruvate dehydrogenase); IDH1/IDH2 (isocitrate dehydrogenase 1, and 2) S-Adenosyl methionine (SAM); S-adenosyl-L-homocysteine (SAH); Nicotinamide adenine dinucleotide (NAD^+^); Nicotinamide adenine nucleotide reduced (NADH); Oxaloacetate acid (OAA).

**Figure 2** shows main intracellular metabolic pathways to provide metabolites for main epigenetic marks (methylation, acetylation, lactylation) and cofactors to modulate the activity of epigenetic enzymes.

## 5 Metabolism and epigenetics deregulation in disease

**Figure 3:**
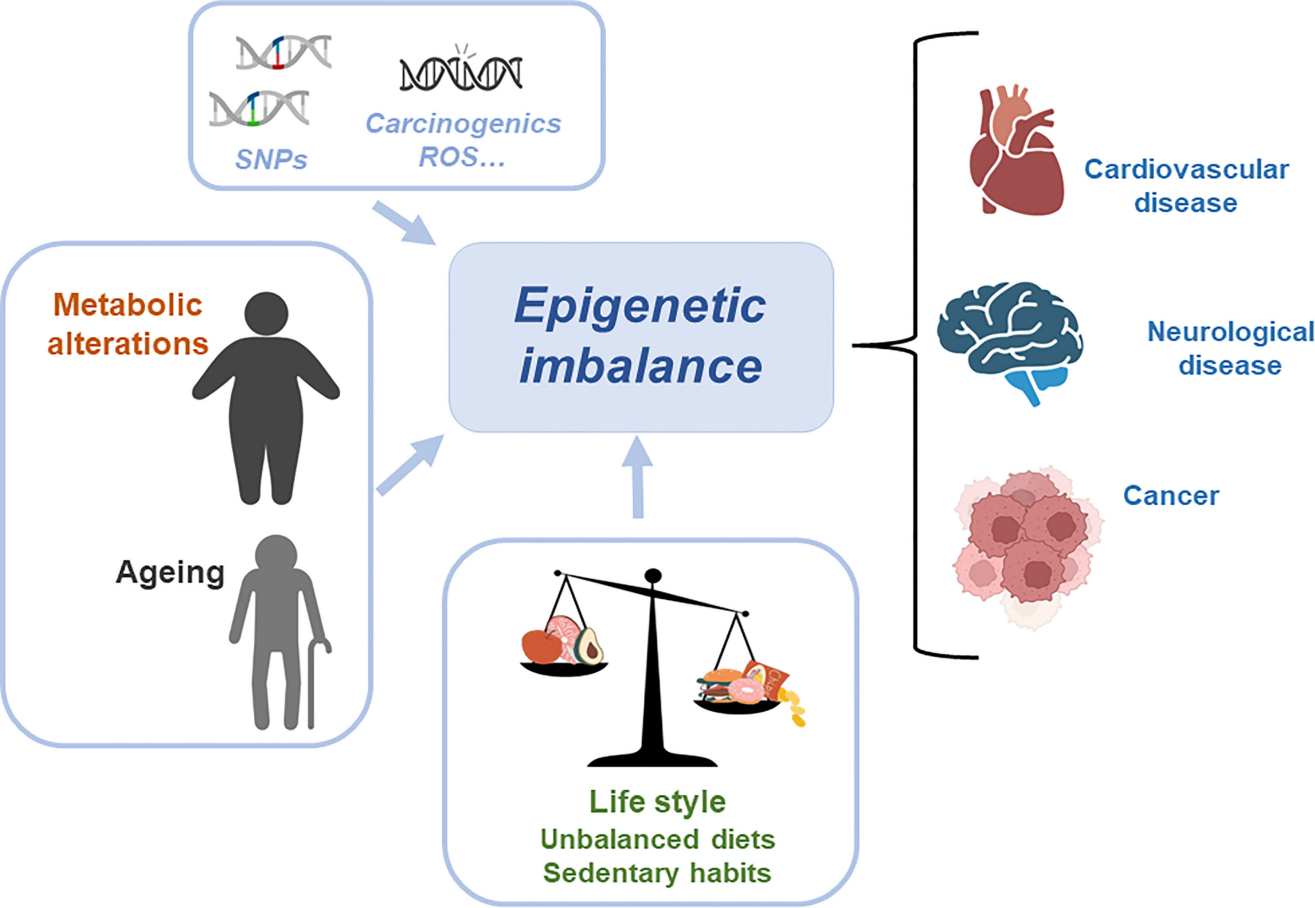
Metabolic and epigenetic imbalances result in disease. There are several factors that can cause an epigenetic imbalance and consequently different diseases. Some of these factors are intrinsic including genetic variations such as Single Nucleotide Polymorphism (SNPs) or DNA mutations caused by carcinogens and Reactive Oxygen Species (ROS); environmental factors including metabolic alterations such as obesity or ageing and lifestyle (unbalanced diet or sedentary lifestyle). This epigenetic imbalance leads to cardiovascular, neurological and cancer diseases.

Metabolic and epigenetic imbalances result in disease. There are several factors that can cause an epigenetic imbalance and consequently different diseases. Some of these factors are intrinsic including genetic variations such as Single Nucleotide Polymorphism (SNPs) or DNA mutations caused by carcinogens and Reactive Oxygen Species (ROS); environmental factors including metabolic alterations such as obesity or ageing and lifestyle (unbalanced diet or sedentary lifestyle). This epigenetic imbalance leads to cardiovascular, neurological and cancer diseases (**Figure 3**).

### 5.1 Genetics influences metabolism and epigenome

Certain genetic variations drive metabolic alterations and thus affect the epigenetic landscape (89). For example, the *C677T* variant in the *MTHFR* gene, which synthesizes 5-mTHF, can impact DNA methylation, depending on the folate status (90). Moreover, this genetic polymorphism has been associated to several alterations including cardiovascular disease (CVD), cancer and neurological diseases (91). Similarly, mutations in the metabolic genes isocitrate-dehydrogenase 1 and 2 (*IDH1* and *IDH2*) are linked to cancer (92), as they increase the production of the 2-hydroxyglutarate metabolite which antagonizes alpha-ketoglutarate (αKG), reduces the activity of DNA and histone demethylases and consequently the DNA and histone methylation status (93, 94). Genetic variants of the fat mass and obesity-associated protein, also known as alpha-ketoglutarate-dependent dioxygenase (*FTO*) gene, which was the first mRNA demethylase identified (95), have been correlated to obesity and metabolic syndrome (96).

### 5.2 Low grade of chronic inflammation affects the epigenetic landscape

In addition to genetic variations, ageing and obesity have detrimental effects on health by different mechanisms that include epigenetic alterations (97, 98).

Obesity is a condition caused by genetic and environmental factors, which is characterized by an increase in body weight, hyperlipidemia, hyperglycemia, hyperinsulinemia, and elevated inflammatory substances. Importantly, obesity increases the risk for other conditions including cardiovascular disease, metabolic syndrome, type II diabetes or cancer (99). At the molecular level, obesity correlates with particular DNA methylation profiles on several genes in different tissues that contribute to its pathophysiology (3, 100). Similarly, RNA methylation misbalances are associated with obesity (101). The expression of m6A regulatory factors in human adipose tissue and Peripheral Blood Mononuclear Cells (PBMCs) varies between lean and obese individuals (102). Thus, the m6A RNA methylomes of genes implicated in lipid metabolism are altered in mice upon a high-fat diet (103). Mechanistically, perturbations in the m6A methylome by FTO (104) or other m6A regulators (105) result in alterations in adipogenesis homeostasis, which likely promote obesity. In addition to alterations in epigenetic factors, the metabolome of obese and metabolically impaired individuals is also unbalanced contributing to epigenetic alterations. For example, a reduction in NAD^+^ levels is linked to metabolic diseases and it is therefore a potential target for intervention in humans (106, 107). In addition, the plasma alpha-ketoglutarate (AKG) levels are reduced upon increasing BMI or HbA1c (a diabetic biomarker), suggesting a possible regulatory role of AKG in pathologies such as obesity. Indeed, AKG regulates JMJD3 expression and promotes the demethylation of *SERPINA1E* promoter, thereby increasing gluconeogenesis (108).

Ageing and age-related diseases (cancer, cardiovascular and neurological diseases) are also a global concern, given their health and economic implications. For this reason, efforts to understand the ageing process have led to the identification of the hallmarks of ageing (97) and the postulation of different theories of ageing (109–112). One of the most deeply characterized epigenetic mark in ageing is DNA methylation, thanks to the development of DNA methylation clocks. DNA methylation profiles correlate to chronological age and clinical variables (113), although their true biological meaning remains obscure (114). Further research is needed to understand how epigenetic age and DNA methylation clocks may help us anticipate, prevent, and overcome diseases related to ageing. Clearly, epigenetic clocks have a big potential as clinical biomarkers (115, 116).

Alterations in RNA methylation are also observed in age-related diseases and, interestingly, m^6^A modifications have been implicated in the regulation of different biological processes associated with ageing, including inflammation (117). Often, these alterations derive from a misbalanced regulation of m^6^A regulatory factors. For this reason, RNA methylation and its biological regulators offer a therapeutic target for age-related diseases.

Further, during ageing, the metabolome profile gets altered, which may contribute to epigenetic alterations. In particular, NAD^+^ levels and AKG levels decline with age (118).

### 5.3 Unhealthy diets have detrimental effects on metabolism and epigenetics

*HFDs* have detrimental effects on systemic metabolic homeostasis. The liver is one of the first organs affected by unbalanced diets. Related to epigenetics, it has been observed that HFDs alter the activity of HDACs and HATs in the liver of rats, diminishing hepatic regeneration and the liver gluconeogenesis which leads to insulin resistance (119). On the contrary, during fasting, the expression of HDACs implicated in the expression of lipogenic enzymes is silenced to favour hepatic gluconeogenesis (120). Furthermore, high-fat diets during pregnancy facilitate the development of metabolic syndrome in the offspring, due to higher levels of histone H3 acetylation (121). This shows the epigenetic implication in the development of metabolic diseases (a type of chronic disease) and also the detrimental and long-term effects of high fat diets.

**Figure 4:**
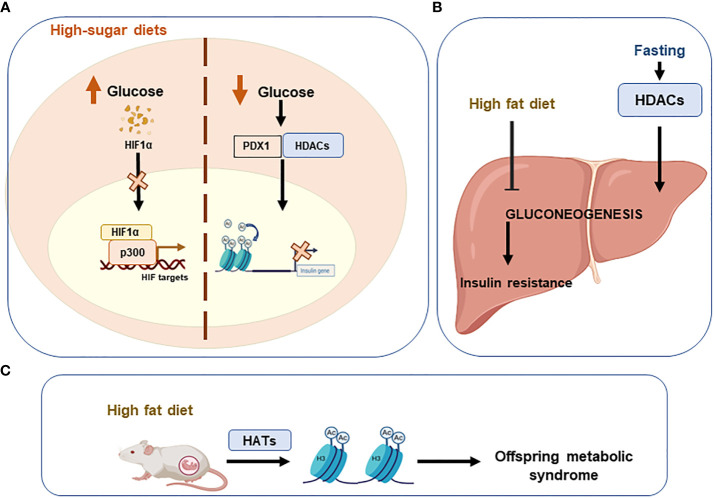
Unhealthy diets leading to epigenetic imbalances. Imbalanced or unhealthy diets have detrimental effects on systemic metabolic homeostasis. **(A)** High sugar diets increase glucose levels, which destabilise hypoxia-inducible factor 1alpha (HIF1A) and consequently inhibit the transcription of HIF. Conversely, when glucose levels are low, acetylation of the insulin gene is promoted to repress its transcription via the PDX1/HDAC interaction. **(B)** A high-fat diet inhibits gluconeogenesis in the liver and promotes insulin resistance. **(C)** Offspring can develop metabolic syndrome if high-fat diets are consumed during pregnancy. Pancreatic And Duodenal Homeobox 1 (PDX1); Histone deacetylases (HDACs); Histone acetyltransferases (HATs).

Lastly, evidence also indicates that *High-sugar diets* augment the risk of metabolic diseases in offspring (122). High glucose levels induce the regulation of insulin transcription by pancreatic β-cells (123). On the contrary, low plasma glucose levels lead to PDX1 (pancreatic duodenal home box 1) binding to HDAC promoting the insulin gene acetylation to repress its transcription (124). The transcription factor HIF1A, which mediates the response of pancreatic β-cells to glucose, is destabilized by high glucose levels interfering its interaction with the coactivator p300 and thus inhibiting the transcription of HIF targets (125) (**Figure 4**).

**Figure 5:**
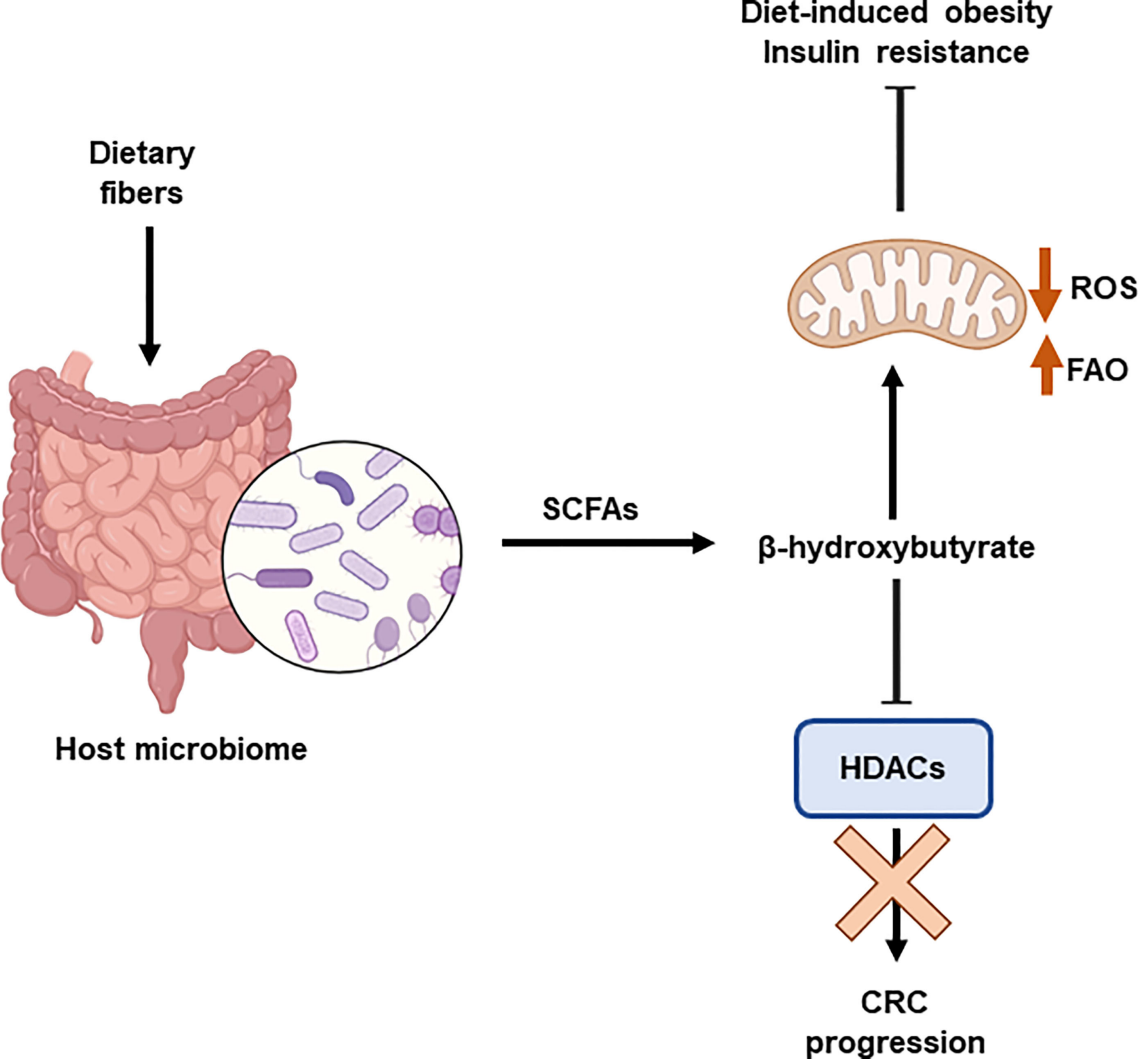
Interplay between epigenetics and microbiome. The host microbiome is influenced by the diet. Dietary fibre induces the production of shirt chain fatty acids (SCFAs), including β-hydroxybutyrate. This metabolite is an HDAC inhibitor that prevents the progression of colorectal cancer (CRC). In addition, β-hydroxybutyrate stimulates mitochondrial function and biogenesis, which protects against obesity and insulin resistance. Histone deacetylases (HDACs); Reactive Oxygen Species (ROS); fatty acid β-oxidation (FAO).

Recently, the interaction between the host microbiome and diet has been shown to influence the epigenetic landscape. Diet modulates the gut microbiome affecting the microbial-produced metabolites and the development of metabolic diseases. The production of short-chain fatty acids, mainly acetate and butyrate, by the microbiome affects the levels of acetyl-CoA and histone acetylation. It has been demonstrated the relevant role of β-hydroxybutyrate, an HDAC inhibitor, in the inhibition of CRC progression in mice by mean of the upregulation of H3 acetylation and the activation of apoptosis. Butyrate is produced by the colonic fermentation of dietary fibers. C57BL/6 mice fed a high-fat diet supplemented with butyrate (5% w/w) consumed more energy than those that did not receive the supplement (126). Butyrate stimulated mitochondrial function and biogenesis in skeletal muscle cells and brown adipocytes, protecting against diet-induced obesity and insulin resistance (Lin et al., 2012). In part, this effect has been demonstrated to be related to the inhibitory effects on HDAC augmenting the expression of FGF21 and fatty acid oxidation (127) (**Figure 5**).

As indicated previously, a large number of miRNAs have been found differently expressed in obese people, suggesting that restoring the systemic metabolic health (mainly glucose and lipid homeostasis) may be partially mediated by miRNAs, as many of them are key modulators of adipogenesis, glycolysis and inflammation (57). Although the list of miRNAs associated with a diet is important, several studies suggest that the miR-17/20/93 family, miR-21/590-5p family, miR-200b/c family, miR-221/222 family, let-7/miR-98 family and miR-203 are the most dysregulated in this context (128, 129).

The expression of miR-21 was found diminished in the WAT of obese humans, correlating with BMI (58). Interestingly, treatment with locked nucleic acid (LNA)-miR-21 treatment reduced weight in a preclinical model of DIO (59). In another study, let-7 knockout mice did not develop insulin resistance after a high-fat diet induced obesity (60).

In a clinical trial with obese women, reduction of body weight showed that circulating levels of several miRNAs were similar to lean controls, suggesting that some of them could be used as biomarkers (61).

## 6 Diet and exercise to counteract epigenetic misbalances

### 6.1 Diet based interventions to modulate epigenetics

**Figure 6:**
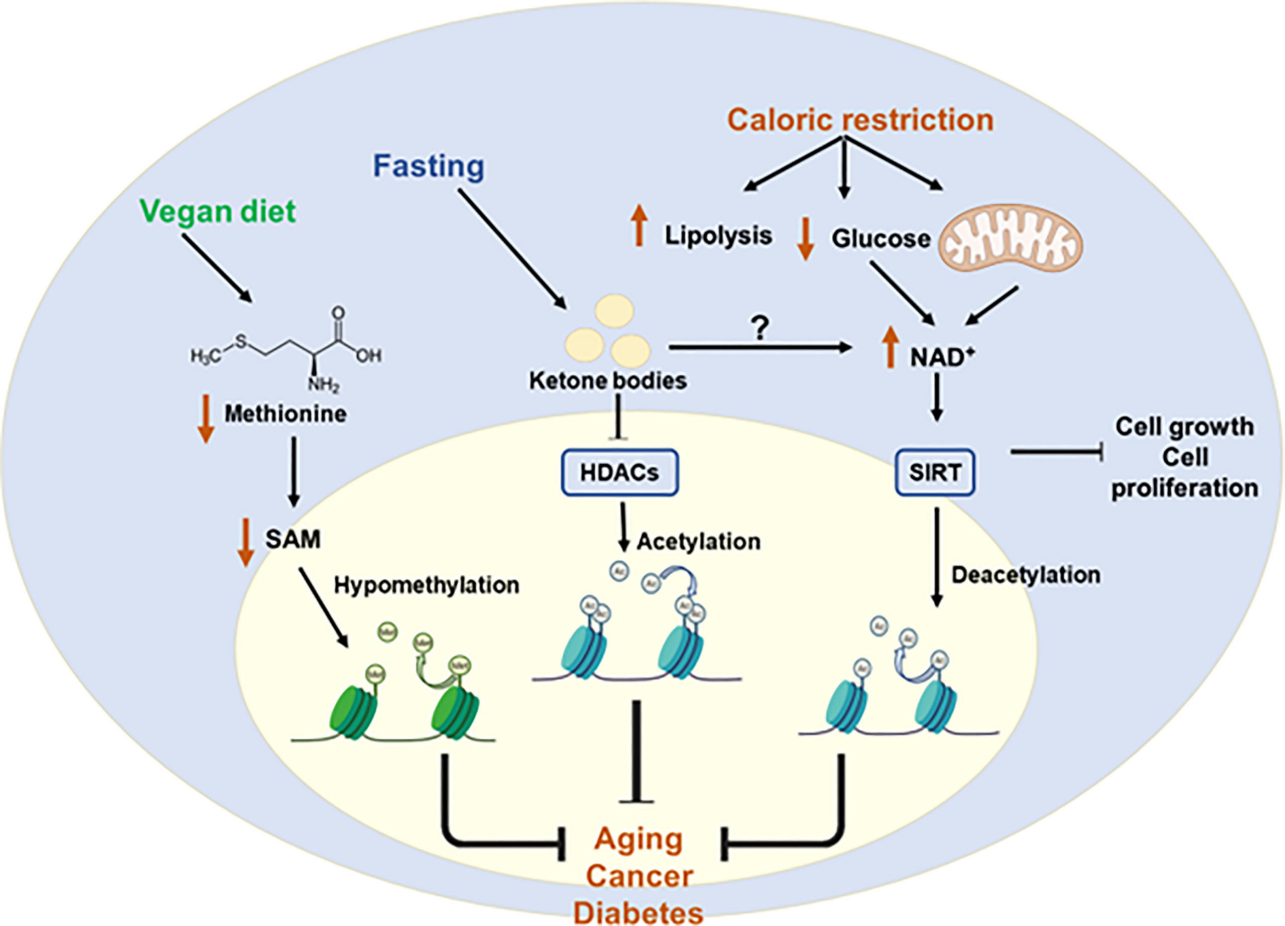
Protective diets to restore epigenetic imbalances. Metabolic diseases caused by epigenetic imbalances can be controlled by protective diets. Caloric restriction increases NAD+ levels by increasing lipolysis and decreasing glucose availability. This increase in the NAD+ leads to the activation of HATs and subsequent deacetylation. Fasting is associated with increased production of ketone bodies which is related to the inhibition of class I HDACs. Finally, a vegan diet is associated with reduced levels of methionine and SAM which leads to hypomethylation of some genes. All these diets may help to counteract chronic and age-related diseases. S-Adenosylmethionine (SAM); Nicotinamide adenine dinucleotide (NAD+); Histone deacetylases (HDACs); sirtuin (SIRT); Histone acetyltransferases (HATs).

Diet is the main source of metabolites -substrates and cofactors- used for the epigenetic programs. Thus, unbalanced diets may affect the epigenome contributing to the development and progression of chronic diseases, as well as longevity. On the other hand, controlling metabolism through nutrient sensing pathways or particular metabolites´ availability may contribute to counteract chronic and age-related diseases. **Figure 6** provides examples of protective diets to modulate epigenetics.

*Calorie restriction (CR)* - reduction of approximately a 20% of the total calorie intake - has been shown to expand lifespan. In mice, the beneficial effects of CR have been associated to the metabolic regulation of epigenetics, although the precise underlying mechanisms are not fully understood. Nevertheless, DNA methylation in specific genomic regions have been described implicated on the beneficial effects of CR on age-related diseases. For example, in the kidney of aged rats, CR was shown to attenuate inflammation, cancer, or diabetes, by restoring the methylation patterns altered during ageing (130). In a similar manner, CR restored liver triglyceride content by mean of epigenetic effects on lipid metabolism related pathways (131). Some of the metabolic adaptations to fasting are associated to the increased production of ketone bodies and/or the inhibition of the class I HDACs, which are implicated in the activation of the expression of metabolic starvation responsive genes (132). A similar effect related to ketogenesis and histone acetylation has been observed during exercise (133). Altogether, diets that limit caloric intake may be efficient to prolong health-span through epigenetic control, thus reducing the impact of age-related diseases. A low-carb ketogenic diet that rescues the hippocampal memory defects in a mouse model of Kabuki syndrome through microbial production of β-hydroxybutyrate, an HDAC inhibitor, leads to changes in H3ac and H3K4me3 in the hippocampus and rescues the neurogenesis and memory phenotypes of these mice models (134). On the contrary, a “Western-type” diet diminishes the microbial SCFAs production, affecting hepatic gene expression by epigenetic mechanisms.

In addition, the regulation of particular *metabolites*´ availability may modulate epigenetic marks. Therefore, dietary interventions that limit or enhance certain nutrients or metabolites may be effective tools to affect the epigenetic landscape of individuals. For example, regulating *methionine* availability through a whole food vegan diet increases longevity in rodents (135, 136). Cancer cells, in addition to the Warburg effect, present a high avidity for methionine which is used to write the epigenetic mark H3K4me3 in specific oncogenic genes (137), and targeting the production of methyl donors from the methionine cycle may provide a therapeutic opportunity in cancer (138), as shown by the inhibition of tumor growth in mice after methionine reduction from diet (139). Other studies have shown the therapeutic benefit of a methionine restricted (MR) diet on the improved systemic glucose and fatty acid profiles and reduction of inflammation and oxidative stress (140). Dietary *choline* is oxidized to betaine by the betaine homocysteine methyltransferase (BHMT), affecting the methionine homeostasis. Disruption of choline/betaine metabolism, by the reduction of *BHMT* gene expression and/or the dietary intake of choline, has been associated with hepatocellular carcinogenesis in animal models (141).

The epigenetic machinery can also be regulated by *vitamins* from diet, including A, B, C and D vitamins (142). Vitamin A is not synthesized by humans and must be obtained from the diet. This vitamin or its derivatives, including retinoic acid (RA), are involved in DNA methylation, histone modification and miRNA regulation. RA inhibits transcription by blocking DNMTs and induces some miRNAs to regulate DNA methylation. Vitamin A can both convert 5hmc to 5mc by altering TET activity and block HDAC while activating HATs (142, 143). B group vitamins are involved in DNMTs activity by being converted to SAM, such as B9, or by acting as cofactors of one carbon metabolism pathway (B6, B3 and B12). Biotin (Vitamin B7) causes transcriptionally repressed heterochromatin formation through the biotinylation process (144). Vitamin C or L-ascorbate is synthesized from ascorbate in mammals and can also be obtained from the diet (145). In relation to epigenetics, this vitamin plays an important role in the demethylation process of DNA and histones in a TET dependent or independent manner, respectively. Like other vitamins, vitamin C can promote the expression of certain miRNAs. For example, vitamin C represses DNMT3A via miR-143 (142, 146). Finally, vitamin D, produced endogenously or ingested with food, represses p21 by methylating its promoter, while enhancing E-cadherin transcription by demethylating its promoter. It is also involved in histone modifications by activating both HAT and HDACs (142, 147).

### 6.2 Exercise and epigenetics

**Figure 7:**
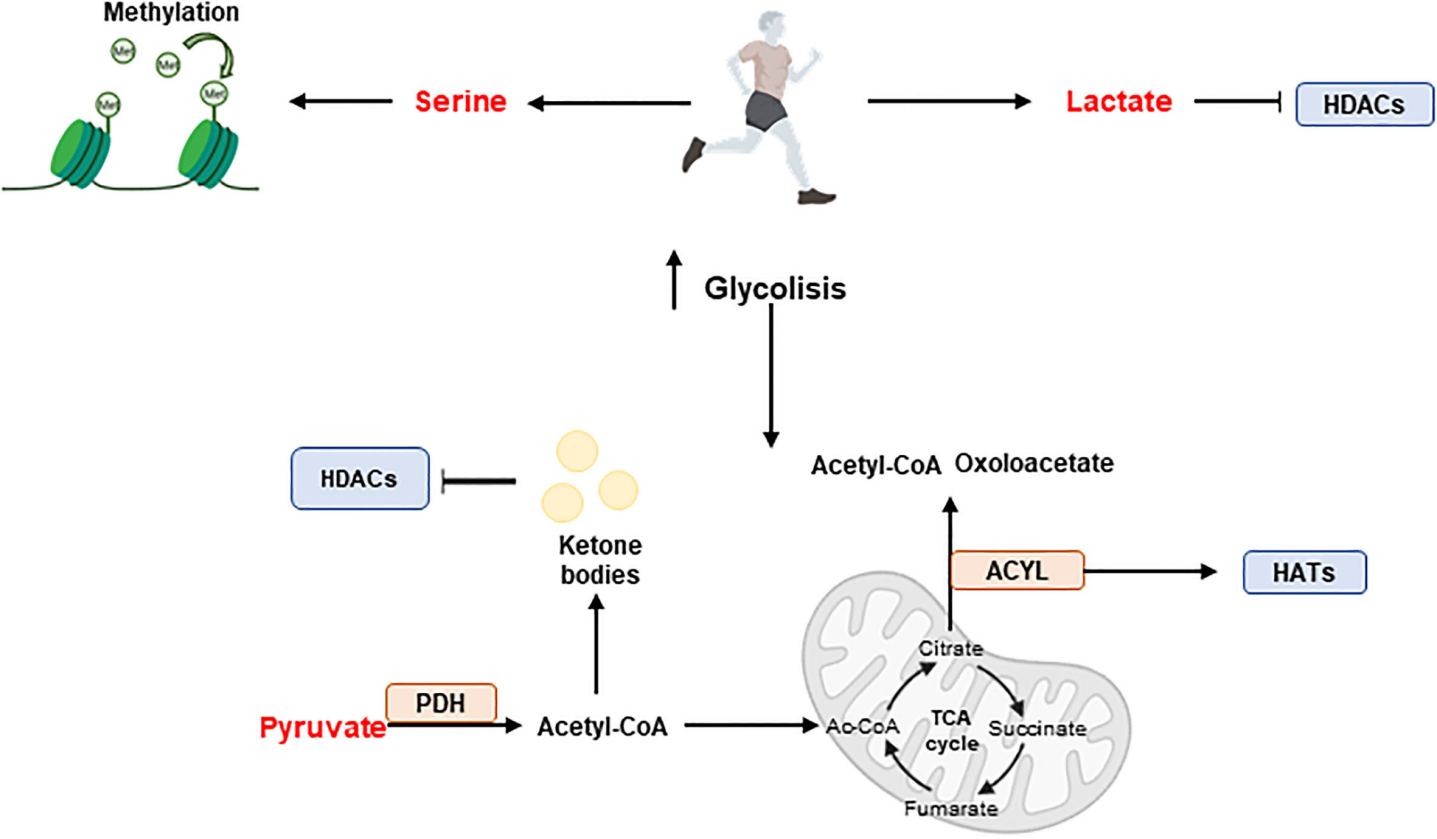
Exercise and epigenetic regulation. During exercise, skeletal muscle produces metabolites such as serine, pyruvate and lactate, which have effects on epigenetics. Serine promotes methylation while pyruvate and lactate have an influence on acetylation. Acetyl-CoA produced from pyruvate enters the tricarboxylic acid cycle (TCA) cycle where key intermediates such as citrate, succinate or fumarate are synthesised. In addition, the ACYL enzyme can produce Ac-CoA and oxaloacetate from citrate and promote hyperacetylation by interacting with HATs. Furthermore, ketone bodies can be formed from acetyl-CoA and participate in HDACs inhibition. Histone acetyl transferases (HATs); Histone deacetylases (HDACs); Pyruvate dehydrogenase (PDH).

Exercise can reverse chronic metabolic diseases by triggering epigenetic modifications (**Figure 7**). For example, exercise augments the methylation of DNA at the regulatory region of *PGC1A* gene promoting fatty acid oxidation and mitochondrial biogenesis in skeletal muscle (148).

In a clinical study, a six-month exercise intervention influenced the genome-wide DNA methylation pattern in human adipose tissue in more than 60 genes related to obesity and insulin resistance (149). In another study, the DNA methylation and miRNA profiles from skeletal muscles in obese and diabetic volunteers were compared at the before and after 4 months of exercise. Interestingly, depending on the type of exercise -aerobic or resistance training- changes in the patterns of DNA methylated genes and miRNAs were observed. Thus, aerobic exercise affected the DNA methylation levels of metabolic genes such as *FASN* and *NRF1*, which correlated with a reduction on the levels of circulating free fatty acids. In addition, miR-29a and miR-132 expression levels were found altered. On the other hand, resistance training augmented the DNA methylation of *SLC2A4*, improving respiratory capacity with a reduction of intramuscular lipid accumulation (150). In another study, acute endurance exercise augmented the expression of miR-1 and miR-133a promoting mitochondrial biogenesis and respiratory capacity (151).

In general, several studies have demonstrated the role of exercise in reducing meta-inflammation in chronic metabolic diseases (152).

During exercise and depending on the intensity, skeletal muscles rapidly metabolize available nutrients -including fats, carbohydrates, and amino acids-, resulting in the production of metabolites such as Ac-CoA, serine, ATP, lactate, butyrate, and α-KG, with effects on the epigenetic programs.

Carbohydrates are the main source of energy during exercise, where glycogen stores are used over muscular triglyceride reserves (153). Upregulation of glycolysis during exercise -high intensity aerobic exercise and resistance exercise- augments the production of metabolites such as serine, pyruvate, and lactate with effects on epigenetics. The upregulation of serine increases the methylation potential in skeletal muscle, meanwhile pyruvate upregulation can further increase the levels of acetyl to produce Ac-CoA. PDH catalyzes the decarboxylation of pyruvate to Ac-CoA during periods of high glucose metabolism. The active form of PDH has been shown to significantly increase almost instantly following exercise - acute aerobic exercise at ranging intensities, and during long term aerobic training- in skeletal muscle (154), suggesting a significant increase in glucose-derived Ac-CoA. Ac-CoA enters the TCA cycle and undergoes a chain of reactions to augment the pool of key intermediates such as citrate, succinate, and fumarate, that have distinct roles in regulating the activity of epigenetic enzymes.

Mitochondrial derived citrate provides nuclear Ac-CoA via a reaction with ACLY, which releases Ac-CoA and oxaloacetate from citrate. In a skeletal muscles ACLY is implicated in myoblast cell differentiation. Recently, overexpression of ACLY has been shown to promote the hyperacetylation of K9, 14, and 27 on histone protein 3, allowing the local concentration of the key myogenic regulatory factor MyoD for the transcription of myoblast cell differentiation genes (155).

Although PDH complex is the main pathway to produce Ac-CoA from glucose, during exercise, the increased demands of energy, specifically in acute and long-term aerobic exercise, induces lipolysis to release FAs for Ac-CoA production through β-oxidation.

Ac-CoA can enter the TCA cycle for ATP production or, during periods of high energy demand, for the synthesis of ketone bodies in the liver, such as acetoacetate and β-OHB, which will be available for energy demanding tissues such as skeletal muscle. During aerobic endurance exercise, fat becomes the dominant energy substrate, and the remaining carbohydrate resources remain intact (156). The cellular mechanisms responsible for this metabolic shift are, however, not yet fully understood. Ketone bodies have important signalling benefits in the skeletal muscle health acting inhibitors of HDAC activity, augmenting the hyperacetylation of histones for the expression genes, such as the forkhead box O3 (Foxo3) (157).

Lactate is an endogenous HDAC inhibitor, sharing common regulatory pathways with SCFA metabolites, such as butyrate and acetoacetate (39). In addition, the epigenetic mark lysine lactylation in the promoter regions of coding genes augments their expression, although the consequences of lactylation epigenetic remodelling during intense exercise requires further research.

### 6.3 Natural bioactive compounds in the regulation of epigenetic enzymes

Bioactive compounds from the diet have been extensively described to modulate the epigenome and contribute significantly to nutritional programs to prevent and/or treat metabolic diseases.

**Table 1** summarizes well known bioactive compounds from diet with health promoting benefits partially mediated by their epigenetic effects.

**Table 1:** Summary of main bioactive compounds implicated in the modulation of epigenetic enzymes.

Class	Subclass	Bioactive compound	Source	Epigenetic Target	Effects	Reference
Flavonoids	Flavonols	Quercetin	Citrus	HAT HDACs DNMTs HMTs	Anti-inflammatory, Anti-cancer	(158–161)
		Kaempferol	Widespread in plant kingdom	HDACs	Anti-cancer, anti-microbial, anti-inflammatory, antioxidant cardioprotective, neuroprotective, and anti-diabetic	(162–164)
		EGCG	Green tea	DNMTs HATs HDACs HMTs	Anti-inflammatory, anti-cancer, antioxidant	(162–164)
	Flavones	Luteolin	Many fruits and vegetables	DNMT HDAC HMT and HAT	Anti-inflammation, antioxidant anti-diabetic anti-cancer anti-bacterial and anti-parasitic	(165–169)
		Apigenin				
	Flavanones	Hesperetin	Citrus fruits	DNMT DOT1L HMTs HDACs	Anti-cancer	(170–173)
		Silibin				
	Isoflavones	Genistein	Soybean	HAT SIRT1 DNMT HDAC NFκB	Anti-cancer, anti-inflammation, antioxidant, anti-microbial and anti-cancer	(174–177)
Non-flavonoids	Stilbenes	Resveratrol	Blue berries and grapes	SIRT1 DNMTs NFκB LSD1 IGF1	Anti-inflammatory, increase insulin sensitivity, cardioprotective, anti-cancer, anti-inflammatory, neuroprotective	(178–189)
	Lignans	Curcumin	Turmeric	HDAC HATs Notch1 NFκB DNMT1 HDAC	Anti-inflammatory, antioxidant, anti-cancer, anti-thrombotic and cardioprotective	(190–192)
Glucosinolates	Isothiocyanates	Sulforaphane	Cruciferous vegetables	HDACs DNMTs	Anti-cancer	(193, 194)
		PEITC				
Thiosulfonates		Allyl mercaptan	Garlic	HDACs	Anti-cancer	(195–197)

The source, targets and effects of each bioactive compound are explained. In red are indicated those targets that are inhibited, in green those that are activated and in orange those that can be both activated o inhibited depending on the cellular context.

#### 6.3.1 Flavonoids

##### 6.3.1.1 Flavonols: quercetin, kaempferol, epigallocatechin-3-gallate

*Quercetin*, a dietary polyphenol present in citrus, activates the NAD-dependent deacetylase SIRT1. In addition, it inhibits the expression of pro-inflammatory genes by mean of the inhibition of the interaction of p300/CBP and post-translational modifications, such as acetylation of H3 histones associated with the promoters of pro-inflammatory genes (158, 159). Quercetin has also been demonstrated to reduce the activity of HDACs, DNMTs and HMTs in a dose-dependent manner in human cervical cancer (160). Through its role in the inhibition of HDAC and DNMT1, quercetin has also been shown to inhibit the cell cycle and induce apoptosis, thus suppressing tumor growth and angiogenesis in preclinical mouse models (161).

*Kaempferol* (3,4′,5,7-tetrahydroxyflavone) is a potential HDAC inhibitor and an anti-cancer agent against many types of cancer (162). Kaempferol stimulates hyperacetylation of histone H3 in HepG2 and Hep3B (hepatoma cancer cell lines) as well as on HCT-116 (colon cancer cell line) (163). Kaempferol induced autophagic cell death via the inhibition of the HDAC/G9a axis and IRE1-JNK-CHOP in gastric cancer cell lines (164).

###### 6.3.1.1.1 Epigallocatechin-3-gallate (EGCG)

EGCG, present in green tea, has been extensively shown to affect the activity of distinct histone modifiers and DNA methyltransferases -DNMT1, DNMT3A, DNMT3B, AURKA, AURKB, AURKC, PRMT6, PRMT7, KDM4A, KDM5C, HDAC5, HDAC6, HDAC7, HDAC11 and UBE2B- activating the expression of various tumor suppressor genes (198). In prostate cancer cells, EGCG has been shown to reduce the activity of class I HDACs promoting the expression of the tumor suppressor genes p21 and Bax (199). EGCG inhibits inflammation by modulating the activity of HATs inhibiting the activity of NF-kB and downstream inflammatory targets (200, 201). EGCG improves insulin sensitivity and reduces obesity by epigenetic mechanisms modulating the muscle function (201). More specifically, EGCG directly inhibits DNMT by interacting with the catalytic site of the DNMT molecule (202).

##### 6.3.1.2 Flavones: apigenin, luteolin

*Apigenin* has been used in studies of many diseases because of its low toxicity and strong biological effects, including anti-inflammation, anti-oxidation, anti-diabetic, anti-tumor, anti-bacterial, and anti-parasitic effects (165). In MB-231 breast cancer cell line, apigenin promoted the acetylation of histone H3 and thus the transcription of *P21WAF1*. Importantly, apigenin inhibited breast cancer tumor growth in a xenograft nude mouse model (166). In human prostate cancer cells, apigenin inhibited HDAC1 and HDAC3, inducing growth arrest and apoptosis (167). Luteolin inhibited the proliferation and metastasis of androgen receptor-positive triple-negative breast cancer cell by mean of the epigenetic regulation -increased H3K56 and H3K27 acetylation- diminishing the AKT/mTOR dependent MMP9 expression (168).

The epigenetic modulatory effects of *luteolin* have been analyzed on HeLa cells, where it modulated the enzymatic activity of DNMT, HDAC, HMT, and HAT to reduce the global DNA methylation resulting in the reactivation of silenced tumor suppressor genes including *FHIT, DAPK1, PTEN, CDH1, SOCS1, TIMPS, VHL, TP53, TP73* (169).

##### 6.3.1.3 Flavonones: hesperetin, silibin

Citrus fruits are rich in aromatic flavonones such as *hesperetin and silibinin*.

*Hesperetin* has been shown to inhibit gastric cancer through epigenetic mechanisms including degradation of DOT1L, which is a Lysine N-Methyltransferase, and thereby reducing histone H3K79 methylation (170). Moreover hesperetin is an activator of SIRT1 contributing to the inhibition of hepatic inflammation via AMPK/CREB pathway (171).

*Silibinin* inhibits the growth of cancer cells, synergizing with DNA methyltransferase and histone deacetylase inhibitors to augment the expression of epithelial markers such as N-cadherin in non-small cell lung cancer cells (172). In human prostate cancer cells (DU145 and PC3), silibinin reduced gene expression levels of EZH2 by increasing H3K27me3 levels, decreased histone deacetylases 1–2 (HDACs1-2) expression levels, and increased total DNMTs activity (173).

##### 6.3.1.4 Isoflavones: genistein

*Genistein* (4’,5,7-trihydroxyisoflavone) is the most abundant isoflavone found in the soybean. Genistein has been shown to mediate post-translational changes in histones. Genistein inhibits SIRT1 leading to increase acetylation of histone H3K9 in *PTEN*, *CYCD*, and *FOXO3A* promoters in prostate cancer cells. Thus, in human prostate cancer cells genistein increases the expression of tumor suppressor genes such as *p21WAF1/CIP1* and *p16INK4a* by regulating chromatin condensation via HAT expression (174). In LNCaP human prostate adenocarcinoma cells, genistein mediates post-translational changes such as the ubiquitination of androgen receptor by modulating the HDAC6-Hsp90 function (175). In prostate cancer cell lines, soy phytoestrogens have been shown to reduce DNA methylation at *EPHB2*, *BRCA1* and *GSTP1* promoters to inhibit cell proliferation and to induce cell death (176). Other genes, such as *hTERT*, *MAD1L1*, *KDM4B* and *TRAF7* have shown reduced methylation profiles after genistein treatment (177).

#### 6.3.2 Non-flavonoids

##### 6.3.2.1 Stilbenes: resveratrol

###### 6.3.2.1.1 Resveratrol

Resveratrol is a polyphenol mainly found in blue berries and grapes. Some studies indicate that the anti-inflammatory effects of resveratrol are mediated by its epigenetic effects on the activity of HDAC11, SIRT1, and p300. Induction of SIRT1 by resveratrol has been demonstrated in several studies, where resveratrol augmented glucose-stimulated insulin secretion in both INS-1E cells (insulin-secreting beta cells) and human islets (178). In another work, resveratrol improved insulin sensitivity in mice fed with a high-fat diet, which seemed to be associated to the increased activity of SIRT1, that repressed the negative regulator protein tyrosine phosphatase 1B (PTP1B) of insulin secretion (179). Similarly, in rhesus monkeys, resveratrol led to an increase in SIRT1 expression and to an improvement in insulin sensitivity in visceral white adipose tissue (180). Many studies suggest that the activation of SIRT1 by resveratrol also contributes to the reduction of the lipid metabolism targets ACC (acetyl-CoA carboxylase), FAS (fatty acid synthase) in human HepG2 hepatocytes exposed to high glucose (181). SIRT1 has been shown to negatively regulate the expression of survivin, a member of the inhibitor of apoptosis (IAP) family, and to play an important role in ageing. Resveratrol has been shown to increase the expression of breast cancer 1, early onset (*BRCA1*), a human tumor suppressor gene, via histone H3 acetylation (182). In prostate cancer cells, resveratrol reduces growth and stimulates apoptosis through activation of FOXO transcription factors. The cardioprotective effects of resveratrol derive from its anti-apoptotic and anti-oxidant effects and its positive modulation of SIRT1 expression (183). In a mouse model of senescent heart, resveratrol improved the cardiac function by mean of its effects on the reduction of Foxo1 acetylation (184). The anti-tumorigenic activity of resveratrol by mean of its epigenetic effects on the HDAC pathway has been demonstrated in tumor models, both *in vitro* and *in vivo* (185, 186). In prostate cancer, resveratrol reactivated the expression of PTEN by abrogating the activity of the MTA1/HDAC complex. In addition, the MTA1 downregulation by resveratrol was shown to be partially mediated through its role in increasing p53 acetylation in PCa cells (187). The role of resveratrol on SIRT1 activation to extend lifespan has been extensively studied. Similar to caloric restriction, resveratrol increases autophagy through SIRT1 activation increasing lifespan both in human cells and *C. elegans* (188). Although several studies suggest that resveratrol is not able to extend lifespan in healthy mice its beneficial effects on metabolic-compromised mammals have demonstrated to be successful (189).

##### 6.3.2.2 Lignans: curcumin

*Curcumin* has been extensively demonstrated to have anti-inflammatory, antioxidant, and anti-cancer effects. During diabetes, high glucose levels break the equilibrium between HAT and HDAC, augmenting the transcriptional activity of NF-kB and it´s downstream pro-inflammatory genes IL-6 and TNF-α. This effect has been shown to be partially reverted by curcumin by mean of its role on the inhibition of HDAC as demonstrated in preclinical mouse models (190). In PC3-M prostate cancer cells, curcumin promoted the proteasome-dependent degradation of p300/CBP without affecting the levels of other HATs such as PCAF or GCN5, being proposed as a therapeutic compound to specifically inhibit p300/CBP HAT (191). In hematopoietic Raji cells, curcumin suppressed the activity of NF-kB and Notch1 proteins, by inhibiting the protein levels of p300/CBP, HDAC13, HDAC1, HDAC3, and HDAC8, contributing to the reduction of inflammatory markers and cell proliferation (192).

#### 6.3.3 Glucosinolates. isothiocyanates: isothiocyanate sulforaphane, PEITC

Cruciferous vegetables are one of the main sources of the *isothiocyanate sulforaphane*, which is a well-known bioactive compound implicated in the induction of phase-II detoxification enzymes. Recently, sulforaphane has been described as an epigenetic modulator in several human diseases, including cancer. Sulforaphane inhibits histone deacetylases HDAC2 and 3, leading to the increase acetylation of histones H3 and H4 on p21 promoter inhibiting breast cancer cell proliferation (193). In LnCaP prostate cancer cells and in breast cancer cells, sulforaphane reduces the expression of the DNA methyltransferases DNMT1 and DNMT3a, contributing to the increased expression of several tumor suppressor genes (194).

*Phenethyl isothiocyanate (PEITC)* has been shown to be an HDAC inhibitor in prostate cancer, leukemia, and myeloma cells (203, 204). PEITC was also shown to inhibit leukemia development in mice. Treatment of LNCaP cells with PEITC inhibited the activity and levels of HDACs and induced selective histone acetylation, whereas in mouse erythroleukemia DS19 cells, allyl isothiocyanate increased histone acetylation without affecting HDAC activity (205).

#### 6.3.4 Thiosulfonates: allyl mercaptan

Several garlic-derived small organosulfur compounds such *as allyl mercaptan* (AM) have been described to inhibit the HDAC activity *in vitro.* In AM-treated human colon cancer cells, HDAC inhibition led to a rapid and sustained accumulation of acetylated histones in total cellular chromatin (195). Chromatin immunoprecipitation assays demonstrated the role of AM on histone H3 acetylation at the *P21WAF1* promoter, favoring the increased binding of the transcription factor Sp3. Interestingly, AM also augmented the binding of p53 in the distal enhancer region of the *P21WAF1* gene promoter, inducing a G1 cell cycle arrest (195). The anti-cancer effects of diallyl disulfide (DADS) and its active metabolite S-allyl mercaptocysteine (SAMC) have been also associated to H3K14 acetylation at the *P21WAF1* gene in colon and breast cancer (196, 197).

## 7 Conclusions

Nutrition, diet, and metabolism regulate epigenetic responses and integrate environmental cues with cellular responses. Healthy diets sustaining balanced epigenetic landscapes have been demonstrated to promote healthspan and to counteract ageing and metabolic diseases. On the contrary, unbalanced diets such as Western diets can alter metabolic and epigenetic traits leading to the development and progression of metabolic diseases and ageing. Diet, exercise, and bioactive compounds from natural sources may provide metabolites and nutrients -vitamins and minerals- to restore epigenetic homeostasis.

## 8 Future directions

Unbalanced diets affect the epigenome contributing to the development and progression of chronic diseases, cancer, and ageing. Therefore, the relationship between lifestyle habits (mainly diet and exercise) and epigenetic regulation is a promising landscape to develop personalized nutrition strategies (nutritional epigenetics). Recently, epitranscriptomics -epigenetic marks in mRNA- is a novel field of research. m^6^A RNA modification has been shown to play a critical role in regulating gene expression and signalling pathways for physiological homeostasis. The molecular interactions between gut microbiota and the metabolo-epigenetic regulation need to be further investigated as part of diagnosis, prognosis, and treatment of human metabolic diseases. (5) In addition, some metabolites have been shown to be affected by circadian clock, establishing a direct link between cyclic rhythms and metabolism in the cell. The circadian clock in cells and the systemic level in a variety of metabolic and physiological processes is in harmony with the external 24-hour cycle of light and dark. Thus, alteration of the circadian rhythm may be associated with the development of NCDs, and ageing through its effects on the cyclic availability of coenzymes and metabolites (206). For example, although the expression levels of SIRT1 are non-cyclic, NAD^+^ synthesis is directly regulated by the circadian clock machinery, which controls the transcription of the *Nampt* gene. These findings suggest that several SIRT1 targets are likely to display circadian oscillations in their acetylation. This is the case for K9/K14 histone H3 sites at circadian gene promoters, as well as BMAL1, a non-histone target of SIRT1, that operates as a transcriptional co-activator of the circadian regulator CLOCK (207).

## Author contributions

MGdC and ARdM contributed to conception and design of the study. MGdC wrote the first draft of the manuscript. RMP wrote sections of the manuscript. All authors contributed to the article and approved the submitted version.
